# Research on Non-verbal Signs of Lies and Deceit: A Blind Alley

**DOI:** 10.3389/fpsyg.2020.613410

**Published:** 2020-12-14

**Authors:** Tim Brennen, Svein Magnussen

**Affiliations:** Department of Psychology, University of Oslo, Oslo, Norway

**Keywords:** lies, lying, deceit, truth, lie detection, non-verbal

## Introduction

Research on the detection of lies and deceit has a prominent place in the field of psychology and law with a substantial research literature published in this field of inquiry during the last five to six decades (Vrij, 2000, 2008; Vrij et al., 2019). There are good reasons for this interest in lie detection. We are all everyday liars, some of us more prolific than others, we lie in personal and professional relationships (Serota et al., 2010; Halevy et al., 2014; Serota and Levine, 2015; Verigin et al., 2019), and lying in public by politicians and other public figures has a long and continuing history (Peters, 2015). However, despite the personal problems that serious everyday lies may cause and the human tragedies political lies may cause, it is lying in court that appears to have been the principal initial motivation for the scientific interest in lie detection.

Lying in court is a threat to fair trials and the rule of law. Lying witnesses may lead to the exoneration of guilty persons or to the conviction of innocent ones. In the US it is well-documented that innocent people have been convicted because witnesses were lying in court (Garrett, 2010, 2011; www.innocenceproject.com). In evaluating the reliability and the truthfulness of a testimony, the court considers other evidence presented to the court, the known facts about the case and the testimonies by other witnesses. Inconsistency with the physical evidence or the testimonies of other witnesses might indicate that the witness is untruthful, or it may simply reflect the fact that the witness has observed, interpreted, and later remembered the critical events incorrectly—normal human errors all too well known in the eyewitness literature (Loftus, 2005; Wells and Loftus, 2013; Howe and Knott, 2015).

When the facts of the case are not well known, witness testimonies, including the testimony from alleged victims, may be critical to a verdict, and these testimonies are sometimes from witnesses who hold a personal stake in the case and shun self-incriminating statements. In many countries, a witness lying in court risks being charged with perjury—the accused typically does not risk such a reaction—but there are still cases where witnesses lie. In such cases, when there is a possibility that one or more of the witnesses are lying and the court's verdict depends upon the perceived credibility of the witnesses, the issue arises of distinguishing between lying and truthful witnesses. Is it possible to identify liars vs. truth tellers based on the non-verbal signals transmitted by the sender?

## What People Believe

Psychological folklore tells us that it is. Studies on what people believe about lying and deceit identify a number of non-verbal cues associated with lying (Vrij, 2000, 2008; The Global Deception Research Team, 2006)—gaze avoidance, fidgeting, restless foot and leg movements, frequent body posture changes. Such beliefs are not restricted to lay persons but held by law and psychology professionals as well (Bogaard et al., 2016; Dickens and Curtis, 2019). Based on such everyday ideas, many countries offer courses and programs that promise lie detection competence. Internationally well-known examples are the SPOT (Screening of Passengers by Observation Techniques) program aimed at identifying possible terrorists at airports by behavior analysis, and the SYNEROLOGY program aimed at disclosing deception in interviewing situations in the courts or in job application interviews (Denault et al., 2020). In our country in 2018, a professional organization which offers advanced courses to members of the legal professions, announced a course called Spot a liar, given by a US professor of law. He “teaches scientifically proven methods to see concealed emotion and detect lies, including how to identify micro-expressions of emotion that last less than a second, recognize when body language reveals lies and when it is meaningless, detect lies in interviews, meetings, investigations, and even over the phone”. Are such ideas supported by empirical research?

## What the Science Tells Us

Several decades of empirical research have shown that none of the non-verbal signs assumed by psychological folklore to be diagnostic of lying vs. truthfulness is in fact a reliable indicator of lying vs. truthfulness (Vrij, 2000, 2008; Vrij et al., 2019). It is a substantial literature. Vrij's (2008) seminal book included more than 1,000 references to the research literature and the recent review by Vrij et al. (2019) identified 206 scientific papers published in 2016 alone. Thus, any reliable non-verbal cues to lies and deceit ought to have been identified by now, *anno* 2020. However, the conclusions drawn by DePaulo et al. (2003), who analyzed 116 studies more than 15 years ago, still appear to be valid. They concluded that “the looks and sounds of deceit are faint,” and the recent review by Vrij et al. (2019) seconded this: “…the non-verbal cues to deceit discovered to date are faint and unreliable and … people are mediocre lie catchers when they pay attention to behavior.” In other words, no reliable non-verbal cues to deception have to-date been identified. The popular Paul Ekman hypothesis of facial micro-expressions as indicators of lies, advertised by many popular courses, has no scientific support (Porter and ten Brinke, 2008). For example, a recent study, which examined the effect of micro-expression training on lie detection and included the presentation of real-life videos of high-stake liars, found that the trained participants scored *below* chance on lie detection, as did the non-trained or bogus-trained participants (Jordan et al., 2019).

It is therefore not surprising that our ability to detect lying vs. truthful witnesses is mediocre. The meta-analysis by Bond and DePaulo (2006), based on a database of more than 25,000 veracity judgments showed that the average score was at chance level (54% correct), and that none of the professions that we might expect to be good lie detectors—police investigators, psychiatrists, interviewers in recruiting companies—scored better than lay persons. Field studies do no better than laboratory studies. Studies of lie detection based on videotaped police interviews with persons suspected of serious crimes, later confirmed guilty (e.g., Mann et al., 2008), do not indicate any differences in the suspect's demeanor between when he is telling a straight lie and when he (later) is telling the truth, and the overall hit rate is not much above chance level. Likewise, studies of TV interviews of mourning relatives of victims of serious crimes begging the perpetrator to come forward, some of whom later turned out to have committed the crime (Vrij and Mann, 2001; Baker et al., 2013), show that truthfulness judgments were close to chance level (for a single exception, see Wright Whelan et al., 2014). A study of routine police controls of cars, some of which had a minor crime to conceal, showed no above-chance level detection of the true crimes (Carlucci et al., 2013). It is therefore not surprising that programs of deception detection based on behavior analysis, aimed at identifying people with concealed malevolent intentions—e.g., terrorists at gate controls in airports—have failed scientific tests (Denault et al., 2020). In fact, a recent and fairly realistic study of the detection of “smugglers” at a border crossing showed that attending to the smugglers' behavior, e.g., signs of nervousness, actually *decreased* the detection performance to below chance (Mann et al., 2020).

## Is There a Future for This Line of Research?

Given the applied aspect of this research—identifying lying or truthful testimonies by individual witnesses—we are obviously not interested in social metrology, in marginally significant relationships obtained in studies with large samples of participants, or only when many factors are included in the analyses (Hartwig and Bond, 2014). Recall that the original promise of the plausible myths about lie detection was that merely observing a witness should be enough to distinguish between liars and truth-tellers. We are looking for signs of deceit that may assist us in everyday life, assist the court when confronted with discrepant witness testimonies in cases where other evidence is sparse or lacking. Due to the clever experimental designs and creative use of real-life situations by highly competent researchers over the last few decades, we have got an answer to the critical question of whether we can detect deceit by looking at peoples' behavior; the answer is *no*.

An analysis by Luke (2019) shows that the literature on cues to deception suffers from several structural problems that cast doubt on even the modest conclusion that some cues to deception have weak support. He shows that there is an issue of huge flexibility in coding of the cues by raters, that there is evidence of selective under-reporting of non-significant findings, and that the literature contains a much higher proportion of significant results than the power of the experiments should lead one to expect to observe. Using Monte Carlo simulations of effect sizes, sample size, and publication bias, he demonstrates the remarkable finding that even if every single potential cue to deception is actually wholly useless one could end up with the scientific literature that we in fact have. Thus, even the cautious conclusion that some non-verbal cues weakly indicate deception is likely to be an overstatement.

Our argument is that research aimed at uncovering non-verbal cues to deception is unlikely to be fruitful. It is simultaneously the case that research on non-verbal cues may well be able to illuminate other issues of basic human communication and interaction, for instance as to what cues people in fact use when making their (poor) judgements of who is lying.

## Discussion

What options does this research field now have? Does one carry on looking for reliable non-verbal cues? Does one concentrate on whether combinations of them are diagnostic of lying? Vrij et al. (2019) suggest that there are grounds for optimism, for instance, by better defining the terms, or by improving measurement of the non-verbal cues. Luke (2019) recommends increasing the power of studies by increasing sample size. We are doubtful that such strategies will be able to provide solace, because they will be unwieldy in the forensic context. To illustrate this, let us consider two phenomena: Vrij et al. (2015) reported that spontaneous saccadic eye movements (a measure related to the widely-believed-but-not-empirically-supported gaze aversion cue) distinguish between truth-tellers and liars, and Mann et al. (2012) reported that if one measures “deliberate eye contact” rather than eye contact *per se*, liars have longer eye contact than truth-tellers. The reason for our skepticism regarding the application of such effects is that it is difficult to apply small (albeit significant) effects to specific instances. With such cues it will generally not be possible to say who is telling the truth at an individual level, or indeed at the level of an individual statement. One could measure the spontaneous saccades of key witnesses or the amount of deliberate eye contact maintained by a witness giving their statement but it is not clear either that such measures are sufficiently reliable, or what the baseline condition should be, against which one would compare the collected data in order to declare the statement a lie or not. Is the research in a blind alley? We believe it is, as far as lie detection in the forensic context is concerned. The idea that governs the research, that there *are* reliable non-verbal signs to lies and deceit is itself an expression of the western psychological folklore—as pointed out by Nortje and Tredoux (2019), the theoretical foundations for the putative non-verbal cues are shaky—and few researchers in the field appear to have fully digested the possibility that the basic premise of their inquiry may be false. For complex intellectual behaviors it has long been realized that there is a number of broad factors that contribute to individual differences—genetics, cultural influences, personal experiences, and situational factors (Engel, 1977, 1980). To complicate matters, a meta-analysis by Bond and De Paulo (2008) showed that participants' truth judgments depended on the sender rather than on the person doing the judging. The effect of sender on veracity judgments has been confirmed in a number of subsequent studies: Some of us appear more (or less) credible than others, independent of whether we are telling the truth or are lying (Porter et al., 2010; Levine et al., 2011; Korva et al., 2013). In addition, the existence of the literature on cultural differences in lie detection, e.g., Castillo and Mallard (2012), would seem to undermine the idea that lies and deceit are in any useful, systematic manner related to behavior on an individual culture-free basis. We may have been looking for a lawfulness in human behavior that exists only in our minds.

Is the rational course simply to drop this line of research? We believe it is. The creative studies carried out during the last few decades have been important in showing that psychological folklore, the ideas we share about behavioral signals of lies and deceit are not correct. This debunking function of science is extremely important. But we have now sufficient evidence that there are no specific non-verbal behavioral signals that accompany lying or deceitful behavior. We can safely recommend that courts disregard such behavioral signals when appraising the credibility of victims, witnesses, and suspected offenders. For psychology and law researchers it may be time to move on.

## Author Contributions

The order of authors is alphabetical. All authors contributed to the article and approved the submitted version.

## Conflict of Interest

The authors declare that the research was conducted in the absence of any commercial or financial relationships that could be construed as a potential conflict of interest.
